# Between support and substitution: the impact of artificial intelligence use on anxiety and learning performance among Spanish majors in China

**DOI:** 10.3389/fpsyg.2025.1710445

**Published:** 2025-11-14

**Authors:** Zigang Wang, Zuoyu Chen, Longxiang Li, Jiamin Sun

**Affiliations:** 1 School of Foreign Studies, University of International Business and Economics, Beijing, China; 2 Department of Spanish, Jilin International Studies University, Changchun, China

**Keywords:** artificial intelligence, Spanish major, anxiety, learning performance, usage behavior

## Abstract

**Introduction:**

Generative AI is increasingly embedded in foreign-language learning, yet its effects may depend on how students use it. We examined how distinct AI usage behaviors relate to learning/career anxiety and learning performance among Spanish majors in China.

**Methods:**

We surveyed 733 Spanish majors from 59 universities (July–August 2025). Instruments captured AI behaviors, learning anxiety, career anxiety, and learning performance. Exploratory and confirmatory factor analyses identified usage dimensions; Pearson correlations and multiple regressions (controlling for gender, age, grade, and program type) tested associations. Mediation/suppression was examined via Bootstrap procedures.

**Results:**

A three-factor structure emerged: (1) substitutive use (AI replaces learners' cognitive work), (2) after-class auxiliary use, and (3) in-class auxiliary use. Substitutive use was positively associated with learning anxiety (β = 0.331) and career anxiety (β = 0.189) and negatively predicted learning performance (β = −0.178). After-class auxiliary use positively predicted performance (β = 0.271) but slightly increased anxiety; both learning and career anxiety exerted small suppressor effects on this positive pathway. In-class auxiliary use showed no significant effects on anxiety or performance. Both learning anxiety (β = −0.221) and career anxiety (β = −0.092) negatively predicted performance.

**Discussion:**

The educational impact of AI hinges on usage patterns and scenarios. Substitutive behaviors elevate anxiety and undermine performance, whereas well-scaffolded after-class auxiliary use benefits performance despite minor anxiety-related suppression. Guiding students to curb substitutive use and optimize auxiliary strategies—especially outside class—may improve outcomes.

## 1 Research background

Since the beginning of the twenty first century, Artificial Intelligence (AI) has gradually moved from laboratories into various sectors of society, becoming a core driving force in the profound transformation of production modes and lifestyles. In the context of foreign language learning, AI tools based on Large Language Models (LLMs) are spreading rapidly: from intelligent dictionaries and grammar-checking software to virtual dialogue partners and personalized learning recommendation systems, AI has quietly become an important assistant for students' autonomous learning in university students. While these technologies expand the temporal and spatial boundaries of learning, they also subtly reshape students' learning methods, emotional experiences, and learning performances.

For foreign language education, the integration of AI represents both a historic opportunity and an unprecedented challenge (Feng and Zhang, 2023). Foreign language majors, as a group most closely connected with language technologies, are particularly sensitive to AI use. Their usage behaviors may exert influence on two levels: on the one hand, AI may alter learning paths and methods, thereby directly affecting learning performance; on the other hand, continuous AI use may also trigger or alleviate anxiety, indirectly shaping learning outcomes through emotional mechanisms. This duality has fueled ongoing debates within academia regarding the value of AI in foreign language learning. Some studies affirm its positive role, regarding it as a “medicine” that enhances learning outcomes by reducing pressure and improving efficiency (Wei et al., 2025). Others, however, highlight its potential “toxicity,” arguing that overreliance on AI may weaken students' linguistic competence and undermine long-term learning (Stone, 2025; Javed, 2024). Nevertheless, most existing research remains at a macro-discursive or case-study level, with relatively few systematic empirical investigations targeting foreign language majors.

Spanish, as one of the six official working languages of the United Nations and the official language of many international diplomatic and economic organizations, surpassed 600 million potential users for the first time in 2024 (Cervantes Institute, 2024). In China's higher education landscape, English has long held a dominant position. However, since the launch of the “Belt and Road” Initiative in 2013, China's cooperation with Latin American countries in trade, culture, and other fields has deepened significantly, leading to a surge in Spanish language education. The number of Chinese universities offering Spanish majors increased from 12 in 1999 to 94 in 2024, reflecting a structural transformation in foreign language education planning from “English dominance” to “multilingual advancement” (Zheng, 2020). Consequently, Spanish majors have become a highly representative group within foreign language education (Xu and Bai, 2017).

Based on first-hand teaching experience, the author has observed that the use of AI tools has become a widespread phenomenon among Spanish majors, while the rationality and actual effectiveness of such applications remain to be empirically validated. These observations provide vivid evidence and highlight the necessity of systematic exploration. In contrast, most current research on the relationship between AI and foreign language learning focuses primarily on English, neglecting equally influential global languages such as Spanish. For example, Pan and Li (2025) examined the role of generative AI in shaping English learners' learning wellbeing, with particular emphasis on the mediating effects of Perceived Teacher Support (PTS) and Growth Language Mindset (GLM). Similarly, Al-Nofaie and Alwerthan (2024) investigated university English teachers' perspectives on using AI tools for micro-course design, providing insights into curriculum sustainability.

In light of this, and to address the limitations of research subject specificity, this study focuses on Spanish majors in Chinese universities. Through empirical methods, it systematically explores the impact and mechanisms of AI use on students' anxiety and learning performance in their professional learning. The study seeks not only to enrich theoretical contributions in the interdisciplinary field of AI and foreign language education, but also to provide both theoretical and practical implications for optimizing learning strategies and improving learning quality in the era of AI.

## 2 Literature review and research hypotheses

The application and penetration of AI in foreign language learning is a complex phenomenon. Its impact on students' anxiety and learning performance must be examined within a broader theoretical framework. Therefore, integrating relevant theoretical foundations with empirical research findings, and clarifying the intrinsic relationships among AI usage behaviors, anxiety, and learning performance, is a necessary precondition for proposing targeted research hypotheses.

### 2.1 Types of AI use

In the context of the deep integration of AI and education, learners' behaviors in using AI tools exhibit a high degree of diversity and cannot be easily generalized. Although no authoritative classification of AI usage has yet been established, the SAMR model in educational technology provides a classic framework for understanding the relationship between technology and learning processes (Blundel et al., 2022). The model differentiates between low-level usage characterized by substitution, and high-level usage characterized by augmentation, modification, and redefinition. Combining with foreign language learning, learners' autonomous use of AI can be similarly categorized into two core types: “substitutive use” and “auxiliary use.”

The defining feature of substitutive use is that AI directly replaces learners' cognitive labor. In this mode, learners delegate tasks that would normally require personal cognitive processing and language practice—such as translation, writing, or answering questions—to AI tools for direct output. Examples include relying on AI to translate texts or generate homework answers in class, or depending entirely on AI to produce communicative content in authentic interactions. In such cases, AI does not function as an external support tool but replaces learners' active engagement with, and internalization of, language knowledge. In contrast, auxiliary use emphasizes AI's collaborative role in supporting, rather than replacing, cognitive processes. Such behaviors typically serve preparatory, processing, or consolidation stages of learning, such as using AI to generate writing ideas and outlines, polish language expression, supplement cultural knowledge, verify answers, organize notes, or resolve doubts in real time. The commonality lies in the fact that learners remain the primary agents of cognitive processing and content production, while AI functions as a tool to enhance efficiency and deepen understanding, without replacing core cognitive processes in language acquisition.

### 2.2 AI use and anxiety

Rollo May, in *The Meaning of Anxiety*, noted that “the apprehension cued off by a threat to some value which the individual holds essential to his existence as a self (May, 1996: 72).” For foreign language majors, performance in coursework and prospects for career development constitute two primary sources of anxiety: present-oriented “learning anxiety” and future-oriented “career anxiety.” This precisely corroborates two of the primary types of AI anxiety identified in the study of Wang and Wang (2022): AI Learning Anxiety and Job Replacement Anxiety. AI use may influence both types of anxiety through a mechanism of “value threat,” not only due to fluctuations in self-efficacy triggered by technological capabilities (Liu et al., 2024), but also because personal use directly exposes individuals to comparisons of competence and risks of replacement, becoming an important approach to experiment the thereat. (Rachman, 1977).

Bandura's social cognitive theory emphasizes that self-efficacy arises from successful personal experiences of capability (Bandura, 1999). Substitutive use, by allowing AI to replace translation, writing, or problem-solving tasks, deprives students of opportunities for independent cognitive processing and ability validation. This absence may foster uncertainty about their own language competence and anticipation of criticism, thereby exacerbating learning anxiety. At the career level, since foreign language majors' competitiveness hinges on linguistic proficiency and cross-cultural communication skills, substitutive use exposes students to AI's advantages in translation speed and content generation. This may reinforce perceptions of “AI being more efficient than humans,” evoke fears of occupational replacement, and ultimately heighten career anxiety. By contrast, the effects of auxiliary use on anxiety may be more complex. Some studies suggest that AI-assisted learning can boost learners' confidence and alleviate classroom anxiety (Zhang et al., 2024) by reducing perceived pressure (Zhang and Li, 2025). However, auxiliary use may still expose students to AI's advantages, potentially increasing perceptions of replacement threats and thereby intensifying career anxiety. Ghotbi et al. (2022) reveal that 65% of students perceive unemployment as the issue most closely linked to AI, reflecting their profound anxiety about AI. Empirical research has also confirmed that the higher the level of AI anxiety, the lower the motivation to learn (Murakami and Inagaki, 2025)

In summary, as (Zhang, 2025) have noted AI exerts a significant influence on learners' affective and psychological variables such as anxiety and enjoyment. A growing body of recent empirical research has further revealed the complexity of this relationship. For instance, Hawanti and Zubaydulloevna (2023) found that AI chatbot-based writing instruction significantly reduced foreign language anxiety among English majors, whereas Ge et al. (2025) reported that frequent exposure to generative AI in learning tasks increased students' “AI-related anxiety.” These studies not only reflect the value-threat mechanism discussed above but also highlight the context-dependent nature of AI's psychological effects. Together, they provide empirical support for the hypothesized associations between AI use and anxiety.

### 2.3 Anxiety and learning performance

Learning performance represents the unity of both process and outcomes, encompassing short-term exam results as well as long-term knowledge internalization and language skill development (Ouyang, 2024). Krashen's *Affective Filter Hypothesis* posits that affective variables such as anxiety and motivation act as a “psychological filter” that impedes the effective absorption of language input (Krashen, 1982). Subsequent empirical studies have confirmed that learning anxiety exerts significant negative effects on learning performance, cognitive engagement, and interaction quality (MacIntyre and Gardner, 1994), making it one of the key emotional factors influencing learning performance. Among various forms of foreign language anxiety, classroom anxiety is especially salient for its inhibitory effects (Yang, 2019). Recent studies have also found negative correlations between foreign language anxiety and learning performance in non-English language majors (Ruan, 2020). Although research on the impact of career anxiety on learning performance is limited, it may operate through mechanisms parallel to those of learning anxiety. Pessimistic expectations about employment and career development can trigger doubts about the value of one's major, which in turn dampens learning motivation and focus (Feng et al., 2015), reducing learning investment and performance (Zhou, 2025). From the perspective of Self-Determination Theory, anxiety undermines students' fulfillment of “autonomy” and “competence” needs (Vallerand, 2000): learning anxiety may induce “fear of making mistakes”, lowering willingness for independent exploration, while career anxiety may erode goal orientation by fostering “doubts about the professional value of their studies”. Together, these factors may reduce sustained academic engagement, ultimately leading to a decline in performance.

### 2.4 AI Use and learning performance

Both Krashen's Input Hypothesis and Swain's *Comprehensible Output Hypothesis* emphasize the importance of “input” and “output” processes in language acquisition. Improvements in foreign language proficiency and performance largely depend on the effective coordination of input and output throughout the learning process (Gu, 2009). Thus, the influence of AI usage behaviors on learning performance essentially hinges on whether they align with this fundamental principle. Substitutive use that bypasses or weakly engages with input-output processes is unlikely to enhance language learning performance and may even be counterproductive. Some studies suggest that reliance on AI to complete tasks directly reduces deep thinking, weakens autonomous analytical skills, diminishes critical thinking ability (Wang, 2025), restricts active exploration of the learning process, and inhibits creativity. As a result, foreign language majors may fail to develop adequate linguistic competence and core professional skills (Zhang and Sheng, 2025), with performance declining particularly in tasks requiring cultural understanding (Cui, 2025). In contrast, auxiliary use may enhance learning performance by optimizing the input-output process in language learning. Recent empirical studies have demonstrated that well-designed AI integration can significantly improve learners' achievement and motivation. For example, Wei (2023) found that AI-assisted language instruction enhanced students' English learning performance, L2 motivation, and self-regulated learning strategies, indicating that properly guided AI use may strengthen both cognitive and affective dimensions of learning. Some scholars have argued that using AI as a feedback tool can significantly improve learners' engagement and revision quality in writing, particularly in terms of wording and structural refinement (Rong et al., 2025). Personalized practice facilitated by AI tools has also been found to positively influence students' basic language skills (Liu et al., 2025) and oral proficiency [British Council and University of Bedfordshire, Centre for Research in English Language Learning and Assessment (CRELLA), 2019]. Moreover, AI's personalized recommendation functions can substantially reduce the time and effort required for foreign language majors to find learning resources and devise study plans, allowing them to concentrate more on the learning process itself (Huang et al., 2024). By improving the precision of input and the effectiveness of output, such behaviors provide critical support for enhancing learning performance. Empirical evidence from recent studies supports this view. Liu et al. (2025) demonstrated that generative AI tools improve students' critical thinking and task performance through personalized feedback, while Zhuang et al. (2025) found that adaptive feedback generated by AI systems significantly enhanced students' writing quality and revision depth in foreign language learning contexts. These findings collectively suggest that the learning outcomes of AI use are contingent upon how the tool is integrated into cognitive processes, emphasizing that the educational impact of AI depends less on the technology itself than on the pedagogy guiding its use.

### 2.5 Research hypotheses

**Figure 1:**
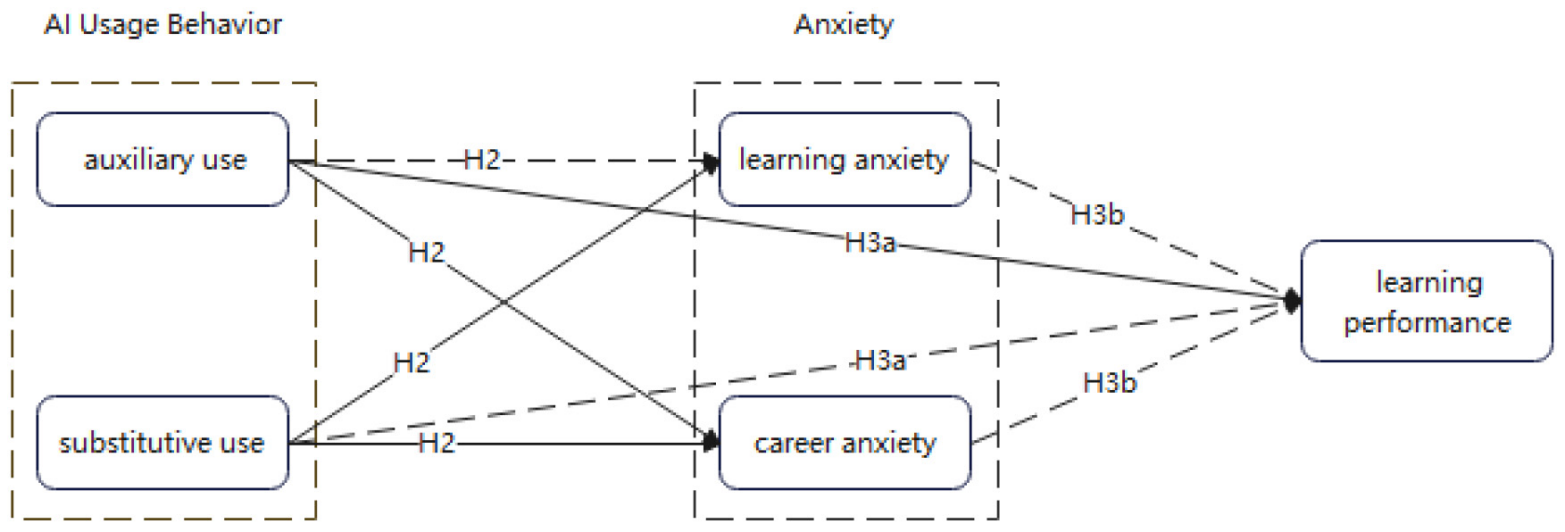
Schematic diagram of the logical relationships among research hypotheses. Solid lines indicate positive effects, while dashed lines indicate negative effects. Drawn by the author.

Based on the above theoretical review and prior empirical findings, this study proposes the following three hypotheses. The logical framework is shown in Figure 1.

H1: AI usage behaviors among Spanish majors consist of two core dimensions: “substitutive use” and “auxiliary use.”

H2: AI usage behaviors exert differentiated effects on anxiety: while substitutive use significantly increases both learning anxiety and career anxiety, auxiliary use reduces learning anxiety but increases career anxiety.

H3: AI usage behaviors influence learning performance through both direct and indirect pathways:

H3a: AI usage behaviors directly affect learning performance, with substitutive use exerting negative effects and auxiliary use exerting positive effects.

H3b: AI usage behaviors indirectly affect learning performance through the mediating roles of learning anxiety and career anxiety.

## 3 Methods and experimental design

### 3.1 Research participants

This study focuses primarily on undergraduate and graduate students majoring in Spanish at Chinese universities, while also including students pursuing double majors and interdisciplinary programs under the “Foreign Language +” framework. The goal is to comprehensively capture the status of AI use across different training models. To avoid the limitations of data drawn from a single institution or region and to enhance the generalizability of the findings, no restrictions were imposed in advance on the type or geographical distribution of participating universities. Instead, participants were recruited nationwide through multiple channels, thereby ensuring the inclusion of students from different grades, genders, and educational backgrounds, and providing a diversified data foundation for subsequent analyses.

### 3.2 Instrument design

Data were collected in two phases using a chain of research instruments that progressed from “behavioral identification” to “quantitative measurement.” The first instrument was an open-ended questionnaire titled *Artificial Intelligence Use Behaviors of Chinese Spanish Majors* (hereafter *Questionnaire (1*), which aimed to systematically document the specific ways Spanish majors employ AI tools. In addition to basic demographic information, participants were required to describe in detail the contexts, methods, and specific behaviors of their AI use in professional learning over the past semester. High-frequency behaviors identified through this process laid the groundwork for subsequent quantitative research. The second instrument was a structured questionnaire titled *Artificial Intelligence Use Behaviors and Their Impacts Among Chinese Spanish Majors* (hereafter *Questionnaire (2*), which served as the core tool of this study. *Questionnaire 2* comprised four sections: 1. Individual Information: Five single-choice items on age, gender, university, grade, and whether the student was in an interdisciplinary Spanish program. These provided the basis for analyzing demographic variables. 2. AI Use Behaviors: This section assessed participants' AI use in their professional learning during the past semester. First, multiple-choice questions identified whether the student had used AI tools and which three were most commonly used. Then, based on behaviors distilled from *Questionnaire 1*, a five-point Likert scale (1 = “very rarely,” 5 = “very frequently”) measured the frequency of typical AI-use behaviors. 3. Anxiety: This section measured both “learning anxiety” and “career anxiety.” Learning anxiety was assessed using the Simplified Foreign Language Classroom Anxiety Scale (Botes et al., 2022), consisting of eight items, which has proven reliable in the Chinese context. Career anxiety was measured using an eight-item scale designed with reference to Zhang and Chen (2006) four-dimensional employment anxiety framework (employment competition, employment support, self-confidence, and employment prospects), adapted in line with Ge et al. (2025). Both scales adopted a five-point Likert format (1 = “strongly disagree,” 5 = “strongly agree”). 4. Learning Performance: This section adapted Lei and Shu (2011) foreign language learning performance scale, comprising four items. The items covered mastery of linguistic knowledge, changes in learning motivation, ability to apply knowledge, and adaptability to learning environments, and were rated on a five-point Likert scale.

### 3.3 Questionnaire administration

*Questionnaire 1* was distributed between June and July 2025, both online and offline, to Spanish majors at five universities. A total of 48 valid responses were collected. By the twent first response, no new behavioral descriptions appeared, indicating saturation had been reached. After excluding non-academic AI use behaviors (e.g., writing novels, casual chatting), the responses were coded, organized, and consolidated, resulting in 14 representative AI-use behaviors (see Table 1). Each behavior was mentioned at least twice.

**Table 1:** AI use behaviors of Spanish Major students in their academic learning.

**No**.	**AI Use Behavior**
1	I ask AI questions when I do not understand the class content.
2	I use AI to take notes or organize key points during class.
3	I consult AI during class to confirm whether my answers are correct.
4	I directly use AI to translate texts or sentences in class.
5	I use AI for interpretation or transcription in class (Chinese–Spanish/Spanish–Chinese).
6	I use AI to generate content directly for in-class questions or exercises.
7	When writing Spanish essays or preparing PPTs, I use AI to generate content.
8	I ask AI to provide inspiration or an outline for Spanish assignments.
9	I use AI to check, polish, or grade my assignments.
10	I use AI to summarize the framework or main ideas of Spanish literature.
11	I use AI to collect supplementary professional knowledge (e.g., terminology, cultural background).
12	I directly use AI to complete written assignments such as translation or fill-in-the-blank exercises.
13	When communicating with Spanish native speakers, I rely on AI for assistance (e.g., writing emails).
14	When listening to Spanish audios or videos outside class, I use AI to transcribe or translate directly.

Drawn by the author.

Questionnaire 2 was completed by the end of July 2025 and distributed in August through the nationwide network of Spanish-language teachers at universities, specifically via course and class groups for Spanish majors. To ensure data quality, the questionnaire included filter questions as well as two attention-check items with designated answers. A total of 900 questionnaires were collected. After excluding invalid responses (non–Spanish majors, failed attention checks, or incomplete answers), 733 valid questionnaires remained, yielding an effective response rate of 81.4%. The sample covered 59 undergraduate institutions across 21 provinces in China. Among the respondents, 77.2% were female and 22.8% male; 95% were undergraduates and 5% were postgraduates. The sample structure largely reflects the demographic characteristics of Spanish majors in China. Reliability testing showed that Cronbach's α coefficient for all scale items in Questionnaire 2 was 0.932 (>0.8), indicating excellent internal consistency, and thus the data can be used for subsequent statistical analysis. During the research process, all participants were informed in advance of the study's purpose and participated voluntarily. For respondents willing to engage in further stages of the study, the research team also collected their contact information, thereby establishing a basis for follow-up in-depth interviews when needed.

## 4 Results and findings

### 4.1 Dimensional structure of AI use behaviors

Among the 733 valid respondents, only two reported not using AI in their professional learning during the past semester, accounting for less than 0.3%. This indicates that AI has been deeply integrated into the learning contexts of Spanish major students. In terms of specific tools, the proportions of students using Deepseek, Cici, and the AI module of Spanish Assistant were 82.5%, 56.6%, and 30.1%, respectively, making them the three most commonly used AI tools in the sample. From the frequency analysis, the median and mean values of the 14 AI use behaviors investigated in this study were both significantly greater than 2, suggesting that these behaviors are not isolated cases among Spanish major students but rather demonstrate a certain level of prevalence and representativeness.

To clarify the dimensional structure of AI use behaviors among Spanish major students, an exploratory factor analysis (EFA) of the 14 items was conducted using SPSS 23.0. The results of the KMO test and Bartlett's test of sphericity indicated that the data were highly suitable for factor analysis (KMO = 0.928 > 0.6; Bartlett's test *p* < 0.001). By extracting factors with eigenvalues greater than 1 and applying Varimax rotation, three factors were identified, with a cumulative variance contribution rate of 64.05%. The factor loadings of each item are presented in Table 2.

**Table 2:** Factor loadings of AI use behaviors among Spanish Major students.

**AI use behavior**	**Factor loadings**			**Communality**
	**Factor 1**	**Factor 2**	**Factor 3**	
I directly use AI to translate texts or sentences in class.	0.707			0.594
I use AI for interpretation or transcription in class (Chinese–Spanish/Spanish–Chinese).	0.647			0.584
I use AI to generate content directly for in-class questions or exercises.	0.742			0.694
When writing Spanish essays/PPTs, I use AI to generate content.	0.651			0.562
I directly use AI to complete written assignments such as translation or fill-in-the-blank exercises.	0.815			0.720
When communicating with Spanish native speakers, I rely on AI (e.g., writing emails).	0.660			0.561
When listening to Spanish audios or videos outside class, I use AI to transcribe or translate directly.	0.748			0.622
I ask AI to provide inspiration or an outline for Spanish assignments.		0.750		0.710
I use AI to check, polish, or grade my assignments.		0.770		0.703
I use AI to summarize the framework or main ideas of Spanish literature.		0.769		0.702
I use AI to collect supplementary professional knowledge (e.g., terminology, cultural background).		0.701		0.539
I ask AI questions when I do not understand the class content.			0.716	0.592
I use AI to take notes or organize key points during class.			0.747	0.639
I consult AI during class to confirm whether my answers are correct.			0.747	0.612

Drawn by the author.

To verify the rationality of the dimensional categorization, confirmatory factor analysis (CFA) was further conducted to assess the model fit. The results showed that the chi-square/degree of freedom ratio of the three-factor model was 1.552 (<2); the comparative fit index (CFI) and the non-normed fit index (NNFI) were both greater than 0.900; and the root mean square error of approximation (RMSEA) was 0.069 (<0.1). All indices met the recommended criteria, indicating a good model fit and confirming the statistical validity of the three-factor structure of AI use behaviors among Spanish major students.

As shown in Table 2, the 14 AI use behaviors were grouped into three different factors, forming the three dimensions of AI use behaviors. Based on the content characteristics of the factors, we labeled them as follows: Factor 1: substitutive use (F1), Factor 2: after-class auxiliary use (F2), and Factor 3: in-class auxiliary use (F3). Substitutive use (F1): This factor mainly includes behaviors in which AI directly replaces students' cognitive effort or learning activities across input, output, and practice processes of language learning. Specifically, it covers classroom reliance on AI for translation, interpretation, and in-class QandA; extracurricular reliance on AI for generating essay content and completing written assignments; as well as using AI to directly produce communicative content in authentic interactions. After-class auxiliary use (F2): This factor primarily includes behaviors in which AI is employed as a learning support tool during self-directed learning outside class, assisting in knowledge construction, content refinement, resource expansion. It encompasses the use of AI for generating writing inspiration, checking and polishing assignments, summarizing the frameworks of literature, supplementing professional knowledge and other practices aimed at constructing and optimizing the knowledge framework. In-class auxiliary use (F3): This factor refers to behaviors in which students use AI as a real-time support tool in classroom learning, assisting in comprehension, note-taking, and knowledge validation. Specifically, it includes consulting AI to clarify misunderstandings, taking notes, organizing knowledge points, and verifying answer accuracy.

### 4.2 The influence of AI use behaviors on anxiety

The correlation analysis was conducted using Pearson's correlation coefficients in SPSS 23.0 to examine the relationships among the three AI use behavior factors (F1–F3) and the two types of anxiety. After testing for multicollinearity, the results of the correlation analysis for all variables are shown in Table 3.

**Table 3:** Correlation between AI use behaviors and anxiety levels (Pearson r).

**Types of AI usage**	**Learning anxiety**	**Career anxiety**
In-class auxiliary use (F3)	0.010	0.034
After-class auxiliary use (F2)	0.060	0.109^**^
Substitutive use (F1)	0.320^**^	0.194^**^

Drawn by the author. ^**^p < 0.01.

The results indicate that Substitutive use was significantly and positively correlated with both learning anxiety (*r* = 0.320, *p* < 0.01) and career anxiety (*r* = 0.194, *p* < 0.01). After-class auxiliary use showed a weaker but still significant positive correlation with career anxiety (*r* = 0.109, *p* < 0.01), while In-class auxiliary use was not significantly related to either type of anxiety (*p* > 0.05).

To further explore the effects of AI use behaviors on students' learning and career anxiety, regression analyses were conducted, controlling for gender, age, grade, and whether the student was enrolled in an interdisciplinary programs. The results are presented in Table 4.

**Table 4:** Linear regression of AI use behavior factors on learning anxiety and career anxiety.

**Variable**		**Learning anxiety**		**Career anxiety**	
		β	***t***	β	***t***
Gender (Male)		0.097^**^	2.707	0.079^**^	2.109
Age		−0.132	−1.962	−0.019	−0.278
Grade		0.014	0.206	0.030	0.418
Interdisciplinary programs (No)		0.043	1.203	0.036	0.965
AI Use Behaviors	Substitutive Use (F1)	0.331^**^	9.150	0.189^**^	5.043
	After-class auxiliary use (F2)	0.071^*^	1.962	0.108^**^	2.853
	In-class auxiliary use (F3)	0.031	0.865	0.060	1.598
*R* ^2^		0.127		0.057	
Adjusted R^2^		0.119		0.048	
*F*		F _(7, 681)_ = 14.214, *p* = 0.000		F _(7, 681)_ = 5.927, *p* = 0.000	
Durbin–Watson		2.032		2.013	

Drawn by the author. ^*^p < 0.05, ^**^p < 0.01.

The results indicated that the substitutive use factor had significant main effects on both learning anxiety and career anxiety, with positive directions. This suggests that higher scores on this factor were associated with higher levels of both learning and career anxiety. The after-class auxiliary use factor also showed significant positive main effects on both types of anxiety, meaning that higher scores on this factor could predict stronger experiences of anxiety. In contrast, the in-class auxiliary factor did not exhibit significant main effects on either learning or career anxiety, indicating that scores on this factor did not have a substantial impact on students' anxiety experiences. Among individual variables, gender displayed a significant positive main effect on both learning anxiety and career anxiety. This implies that, compared with male students, female Spanish majors tended to report higher anxiety levels.

In terms of effect size, the substitutive use factor exerted the strongest main effects on anxiety experiences (β = 0.331 and β = 0.189). This indicates that, regardless of whether learning anxiety or career anxiety is considered, this factor played a greater role than the other variables examined, making it a key determinant of students' anxiety experiences.

### 4.3 The Influence of AI Use Behaviors on Learning Performance

Considering that learning anxiety and career anxiety may act as mediating variables between AI use behaviors and learning performance, the research team first tested the total effects of the three AI use behavior factors on learning performance, with learning performance as the dependent variable (Model 1). Subsequently, learning anxiety and career anxiety were added as independent variables to examine their significance in predicting learning performance (Model 2). The results are presented in Table 5.

**Table 5:** Linear regression of AI use behavior factors on learning performance.

**Variable**		**Model 1**		**Model 2**	
		β	***t***	β	***t***
Gender (Male)		−0.056	2.707	−0.028	−0.806
Age		0.048	0.723	0.017	0.261
Grade		0.054	0.804	0.061	0.939
Compound Spanish Major (No)		−0.021	−0.587	0.006	0.965
AI Use Behaviors	Substitutive Use (F1)	−0.269^**^	−7.525	−0.178^**^	−4.853
	After-class auxiliary use (F2)	0.245^**^	6.782	0.271^**^	2.853
	In-class auxiliary use (F3)	0.055	1.547	0.066	1.936
Learning Anxiety		–	–	−0.221^**^	−5.600
Career Anxiety		–	–	−0.092^*^	−2.431
R^2^		0.150		0.214	
Adjusted R^2^		0.141		0.204	
*F*		F _(7, 677)_ = 17.027, *p* = 0.000		F _(9, 675)_ = 20.397, *p* = 0.000	
Durbin–Watson		2.028		2.013	

Drawn by the author. ^*^p < 0.05, ^**^p < 0.01.

The regression results of Model 1 indicated that the substitutive use factor and the after-class auxiliary use factor had significant total effects on learning performance, with the former being negative (β = −0.269) and the latter positive (β = 0.245). The in-class auxiliary use factor did not exhibit a significant total effect. Results of Model 2 showed that both learning anxiety and career anxiety had significant negative main effects on learning performance (β = −0.221, *p* < 0.01; β = −0.092, *p* < 0.05). Compared with Model 1, after controlling for the two anxiety variables, the main effects of substitutive use and after-class auxiliary use remained significant (β = −0.178, *p* < 0.01; β = 0.271, *p* < 0.05), though the effect size of substitutive use decreased (0.269 > 0.178) while that of after-class auxiliary use increased (0.245 <0.271). Considering the significant positive main effects of substitutive use and after-class auxiliary use on learning and career anxiety identified in the previous analysis, it can be preliminarily inferred that the two anxiety variables may serve as partial mediators or suppressor variables in the relationships between these two AI use behavior factors and learning performance.

To further examine the presence of such effects, the research team employed the Bootstrap method, which is currently considered an optimal approach for testing mediation (Wang, 2014). This method repeatedly resamples the original data to calculate the mediation effect coefficients and their proportion of the total effect, and tests the significance of the mediation coefficients through confidence intervals. The results of this analysis are presented in Table 6.

**Table 6:** Mediation effect test results of AI use behavior factors.

**Path**		**Test conclusion**	**Total effect**	**Mediation effect**	**Direct effect**	**95% CI**	**Effect proportion**
Substitutive use	Learning anxiety	Partial mediation	−1.194	−0.326	−0.788	−0.110 ~−0.040	27.27%
	Career anxiety			−0.081		−0.038 ~−0.002	6.75%
After-class auxiliary use	Learning anxiety	Suppression effect	1.084	−0.072	1.202	−0.038 ~ 0.001	5.95%
	Career anxiety			−0.047		−0.024 ~ 0.000	3.88%
In-class auxiliary use	Learning anxiety	Not significant	0.244	−0.027	0.295	−0.024 ~ 0.011	0%
	Career anxiety			−0.023		−0.017 ~ 0.002	0%

Drawn by the author.

The findings indicate that both learning anxiety and career anxiety play a partial mediating role in the relationship between substitutive use of AI and learning performance, accounting for 27.27% and 6.75% of the total effect, respectively. This suggests that the impact of substitutive use on learning performance is partly realized indirectly through anxiety levels. By contrast, in the path from after-class auxiliary use to learning performance, learning anxiety and career anxiety exhibit a suppressing effect, with effect ratios of 5.95% and 3.88%. This implies that the effect of after-class auxiliary on learning performance is partially “offset” by the anxiety. In the case of in-class auxiliary use, the mediating role of anxiety is not significant.

**Figure 2:**
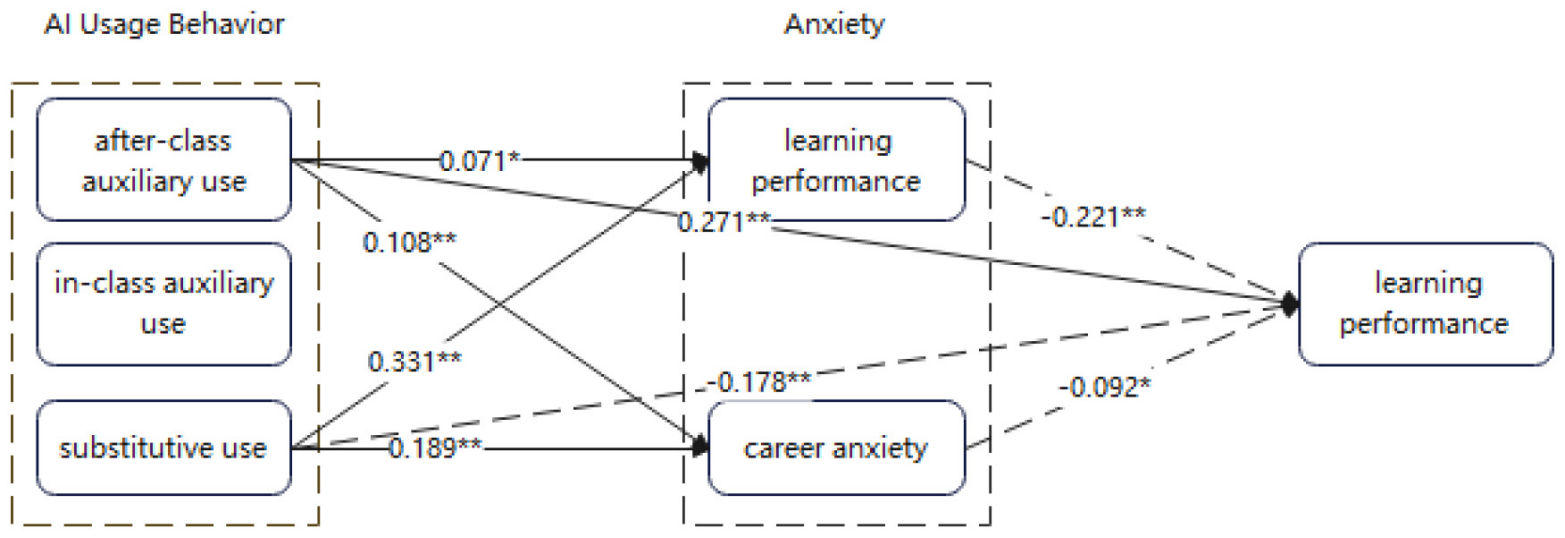
Effects of AI usage behaviors on learning performance. Compiled by the author. Solid lines represent positive effects; dashed lines represent negative effects. Drawn by the author.

Taken together with the regression results in Tables 4, 5 the mechanism through which AI usage behaviors influence learning performance among foreign language majors can be further illustrated in Figure 2.

## 5 Analysis and discussion

The findings further confirm that artificial intelligence has become deeply embedded in the everyday learning practices of Spanish majors in Chinese universities. Data show that generative AI tools such as Deepseek, Cici, and the AI module of Spanish Assistant have become the primary aids for this group, with only 0.3% of students reporting that they had never used AI in their professional studies. In terms of behavioral patterns, students' engagement with AI demonstrates both comprehensive coverage across contexts and diverse modalities: AI use extends throughout in-class learning and after-class self-study, and spans all core language-skill domains including speaking, listening, writing, and reading. This extensive penetration suggests that AI is no longer a supplementary option in foreign language learning, but is increasingly a central component of the daily learning ecology. Notably, statistical analyses controlling for individual-level variables reveal that, apart from gender—which showed significant differences in anxiety—factors such as age, grade, and program type (single vs. interdisciplinary major) had no significant impact. This finding underscores the central role of AI usage behaviors in shaping students' learning experiences. To further illuminate the underlying mechanisms of these quantitative results, the research team conducted follow-up interviews with volunteers who had provided contact information. The qualitative data provided richer insights and validated the empirical findings. Based on both quantitative and qualitative evidence, the verification of the research hypotheses is summarized below.

### 5.1 Types of AI usage behaviors: binary classification, three-dimensional structure

The findings overall support Hypothesis 1 (H1) and further refine the typological features of AI usage. Exploratory factor analysis identified 14 representative AI usage behaviors among Spanish majors, which clustered into three distinct but related dimensions: substitutive use, in-class auxiliary use, and after-class auxiliary use.

The substitutive use factor aligns closely with the pre-defined binary classification: it essentially involves delegating learning outcomes directly to AI, thereby replacing individual cognitive effort. This spans both classroom and after-class contexts, such as relying on AI for translation, assignment completion, or real-time QandA. In such cases, students bypass direct activation of language skills and knowledge construction, positioning AI as a “learning surrogate”. By contrast, in-class auxiliary use and after-class auxiliary use together constitute the broader category of auxiliary use, consistent with the hypothesized framework. Here, students remain the main agents of content processing and production, with AI serving as a collaborative tool to optimize efficiency, deepen understanding, or enhance knowledge absorption, without replacing core cognitive processes.

Importantly, the study reveals that auxiliary use itself bifurcates along contextual lines: in-class auxiliary use emphasizes real-time support (e.g., instant clarification, note-taking, answer verification), while after-class auxiliary use emphasizes deeper learning support (e.g., generating writing frameworks, polishing assignments, or supplementing professional knowledge). This scenario-driven differentiation indicates that, even within the same category of auxiliary use, the connotations and functional positioning of in-class and after-class behaviors differ significantly. Scenario characteristics play a key role in shaping the meaning and effectiveness of AI-assisted behaviors. To some extent, this finding enriches the typological research on AI use behaviors and provides a more nuanced perspective for analyzing the adaptation mechanisms between technological tools and foreign language learning contexts.

### 5.2 AI usage behaviors and anxiety: overall elevation, differential effects

The findings partially support Hypothesis 2 (H2). Substitutive use significantly increases both learning anxiety and career anxiety, consistent with expectations. However, the effects of auxiliary use diverge from predictions—after-class auxiliary use slightly increases career anxiety and significantly heightens learning anxiety, while in-class auxiliary use shows no significant relationship with either form of anxiety.

The strong positive effect of substitutive use supports the “value-threat” explanation of heightened anxiety, while its effect being significantly greater than that of other influencing factors reveals the critical role it plays. Interviews with high scorers on this factor revealed that substitutive use eroded students' sense of self-efficacy. Although tasks were formally completed, many experienced feelings of inadequacy, exacerbating learning anxiety. As one student reported: “*When I let AI solve problems, I can't really follow its logic or framework. I just feel frustrated, and it makes me more anxious.”* Professionally, repeated exposure to AI's advantages in translation and content generation reinforced the perception that “AI is more efficient than humans”, intensifying doubts about personal competitiveness and sparking “career substitution threats”. As observed, most respondents expressed a clear belief that artificial intelligence will replace traditional language-related jobs. One interviewee frankly admitted: “*AI does it so much better than I do. My language level is just average… in the future, it'll be really hard to find a job or advance my career. I feel anxious about that.”*

The finding that after-class auxiliary use elevates learning anxiety was unexpected. Although its effect is clearly weaker than that of “substitutive use,” we argue that, to some extent, it may still stem from the value-threat experience linked to self-assessment and perceived performance. Follow-up interviews with students who scored high on the “after-class auxiliary use” factor revealed that when learners used AI to inquire about knowledge points, polish assignments, generate writing frameworks, or summarize literature, they were directly exposed to AI-produced “high-quality exemplars.” Comparing the “AI standard” with their own abilities often amplified their perceived gap between “ideal competence” and “actual competence,” thereby reinforcing negative self-evaluations of “failing to meet learning requirements” and triggering learning anxiety. As one interviewee noted, “*When I used AI to look up Spanish slang, I felt that its explanations were even more precise than my teacher's slides, which gave me a strong sense of futility in my learning.”* The slight increase in career-related anxiety associated with such behaviors may originate from students' tendency to generalize AI's efficiency advantages in after-class learning tasks to the professional sphere, leading to concerns about “*basic language tasks being replaced*”. However, since AI functions only as a collaborative tool in “after-class auxiliary use,” its impact on anxiety is significantly weaker than that of “substitutive use.” Indeed, many respondents emphasized that they still believe they will retain a certain degree of competitiveness in the future job market.

The non-significant effect of “in-class auxiliary use” on anxiety may be attributed to the neutral and balanced nature of threat perception in classroom contexts. Interviews with participants who scored high on the “in-class auxiliary use” factor revealed that, on the positive side, AI's functions of instant clarification and answer verification helped reduce temporary anxiety caused by “answer uncertainty” and enhanced a sense of security in classroom participation, aligning with the expected value of an auxiliary tool. However, on a more implicit level, frequent reliance on AI for note-taking or translation could divert attention and heighten concerns about one's own learning performance. As one respondent explained, “*Sometimes when the teacher is explaining a knowledge point, I am still using AI to review the previous one, which makes me feel embarrassed and conflicted… and when the teacher seeks feedback, I get nervous.”* To some extent, it is precisely this offset between positive effects and hidden costs in the classroom scenario that prevents significant fluctuations in anxiety levels. At the professional level, such behaviors focus primarily on classroom knowledge comprehension rather than comparisons involving occupational skill substitution. For users, AI in this context mainly serves as a “temporary tool” providing mechanical support, and therefore seldom triggers anxiety related to threats to one's core value.

### 5.3 AI usage behaviors and learning performance: dual effects, two pathways

The findings partially support Hypothesis 3 (H3). Substitutive use exerted a negative direct effect and a mediated effect via anxiety on learning performance, while after-class auxiliary use showed a positive direct effect but a suppressing effect via anxiety. In-class auxiliary use had no significant effect on performance. This differentiated result confirms the important role of the way AI is used in influencing learning performance.

The significant negative impact of “substitutive use” on students' learning performance suggests that such practices may “bypass” or even “disrupt” the processes of language learning input and output. As revealed in follow-up interviews with students scoring high on the “substitutive use” factor, frequent reliance on AI to directly generate outputs—skipping core cognitive steps such as vocabulary discrimination, knowledge retrieval, and feedback-based revision—often results in hollowed learning outcomes, with knowledge points failing to be internalized and absorbed, ultimately leading to declines in performance. Interviewees' accounts vividly reflect this pattern: “*After relying on AI to finish assignments, I still couldn't write anything when facing the same questions in exams”; “Using AI just to cope with homework means I definitely haven't truly learned anything”; “After using AI, I didn't really think through the problems… I can't guarantee I've mastered them.”* Furthermore, the mediating effects of learning anxiety and career anxiety may drive a vicious cycle: weakened competence induces anxiety, which, through the “affective filter,” impedes the intake of new knowledge, while negative career expectations further undermine motivation, compounding performance decline.

Although not all forms of “auxiliary use” necessarily enhance learning performance, the positive effects of “after-class auxiliary use” remain noteworthy. Follow-up interviews revealed that functions such as AI-assisted proofreading and literature summarization reduce information-filtering costs, enabling students to focus on internalizing core content and thereby facilitating a virtuous cycle of input–feedback–revision–output. As respondents expressed, after-class AI use felt like “*asking the teacher questions at zero cost”* or “*After completing exercises, I immediately used DeepSeek to correct mistakes while the memory was fresh and asked it to explain why. This often gave me a sudden sense of clarity… afterward, I rarely repeated the same mistakes.”* Such convenient, instantaneous, and targeted feedback directly supports the synergy between input and output in foreign language learning, ultimately boosting performance. Nevertheless, the suppression effect of anxiety also deserves attention, as it partially offsets the positive impact of after-class auxiliary use, preventing its full potential from being realized.

“In-class auxiliary use,” by contrast, showed no significant effect on learning performance, once again underscoring the classroom's unique role as a setting for AI-assisted learning. While AI's immediate support can help students better understand lecture content and take notes, the classroom is also the core site for teacher–student and peer interactions. Excessive in-class auxiliary use may compromise listening quality, reduce learning engagement, and undermine interaction and real-time feedback absorption. As one respondent observed, classroom AI use can shorten the time spent engaging with problems or knowledge points, leading only to a superficial grasp: “*Sometimes when I was still stuck on the first topic with AI, the teacher had already moved on to the third slide… it was hard for my mind to keep up.”* In some cases, AI's efficiency even fostered a “*take-it-or-leave-it”* attitude toward classroom learning. Comparative analysis further showed that students with frequent in-class auxiliary use were more likely to fall into the bottom 25% of final exam rankings—a pattern not observed for other AI use behaviors—providing indirect evidence that such practices may implicitly undermine learning outcomes.

## 6 Conclusion and reflection

This study explored how different patterns of artificial intelligence (AI) use influence anxiety and learning performance among Spanish majors in China. Drawing on questionnaire data collected nationwide from 733 respondents, the findings reveal a dialectical relationship between support and substitution in AI-assisted language learning. AI itself possesses neither inherent “toxicity” nor “therapeutic value.” Its influence on anxiety and learning performance ultimately depends on students' usage patterns and the fit between the tool and the learning context. When AI is used as a direct substitute for the core cognitive labor of language learning, it undermines and disrupts the process of foreign language acquisition, leading to a significant decline in learning performance. At the same time, it weakens students' self-efficacy, magnifies perceptions of substitution threats, and heightens both “near-term worries” about learning performance and “long-term concerns” about career development, thereby reinforcing the negative impact on learning outcomes. In such usage contexts, AI functions more as a substitutive burden than as genuine support. Conversely, when AI is employed in after-class settings to support foreign language knowledge learning and efficiency improvement, it can, through personalized feedback and expanded resources, act as an effective learning support that enhances performance—although its positive effect may be partially offset by the anxiety arising from students' perceived competence gaps. As for AI-assisted usage in classroom settings, our findings suggest a balance between positive support and hidden costs: it produces no significant effects on anxiety or performance, and thus functions more like a “neutral tool.” These findings empirically demonstrate the value and mechanisms of AI in Spanish-major learning and offer practical guidance for educators in helping students adopt AI tools in a more regulated and constructive manner.

More specifically, the practical implications of this study can be summarized in four aspects: 1. Establishing a clear behavioral-cognitive framework. Teachers should help students systematically identify the types of AI usage in academic learning, with special attention to distinguishing “substitutive use” from in-class and after-class “auxiliary use”. Through typical case analyses, students can better understand the impact mechanisms of different behaviors on ability development and build a “behavior–impact” cognitive association. 2. Strictly curbing substitutive use. Beyond explicitly prohibiting it in teaching regulations, it is necessary to optimize assessment systems to push behavioral change—by increasing the proportion of process-based assignments, reinforcing in-class real-time questioning, and implementing ability-verification mechanisms—so that students cannot rely on AI shortcuts and are compelled to engage in autonomous cognitive processing. 3. Scientifically guiding after-class auxiliary use while regulating anxiety. Teachers can design task templates tailored to core Spanish skills (listening, speaking, reading, writing, and translation), guiding students to use AI for instant feedback and cultural knowledge expansion. Meanwhile, by explaining the limitations of AI feedback in class and organizing sharing sessions on “self-growth with AI assistance,” teachers can alleviate anxiety stemming from perceived competence gaps and release the positive potential of auxiliary use. 4. Taking a balanced view of in-class assistive usage. Teachers need not impose outright bans, but should design pedagogical interventions that balance technology support with classroom participation—for instance, adding “pause AI” reminders at key points in presentations, using frequent real-time questioning to maintain active listening, and holding regular reflection activities where students share both benefits and distractions of AI usage. Such strategies cultivate self-regulation and ensure that technology serves core instructional goals.

Although this study provides new empirical evidence on how different types of AI use behaviors influence anxiety and learning performance among Spanish majors, several limitations should be acknowledged. First, the sample was restricted to Chinese university students majoring in Spanish, which limits the generalizability of the findings across other language contexts. Future research could adopt a comparative approach to examine whether the observed relationships hold for learners of other foreign languages, such as English or German. Differences in linguistic distance, exposure to authentic environments, and cultural familiarity may moderate both the psychological and behavioral responses to AI use. Expanding the scope of comparison would help to clarify the cross-linguistic mechanisms through which AI affects language learning and anxiety. Addressing these questions will not only deepen our theoretical understanding but also inform more adaptive pedagogical strategies in the AI-driven learning environment.

In today's rapidly evolving educational ecosystem, the key issue in Spanish-major teaching is not rejecting AI but building a rational understanding of “technology as a tool serving ability development.” Only through scientific guidance that restores AI to its role as an “assistant” can it truly empower language acquisition and help relieve learning stress. This requires educators to uphold a learner-centered principle in technology use: leveraging AI to improve learning efficiency while remaining alert to risks of ability substitution. Ultimately, this approach enables a positive coexistence of technological efficiency and humanistic value in professional education, paving a sustainable path for high-quality talent cultivation in foreign languages in the AI era.

## Data Availability

The raw data supporting the conclusions of this article will be made available by the authors, without undue reservation.
